# 1,8-Cineole Ameliorates Advanced Glycation End Products-Induced Alzheimer’s Disease-like Pathology In Vitro and In Vivo

**DOI:** 10.3390/molecules27123913

**Published:** 2022-06-18

**Authors:** Fengmao An, Yuhan Bai, Xinran Xuan, Ming Bian, Guowei Zhang, Chengxi Wei

**Affiliations:** 1 Institute of Pharmaceutical Chemistry and Pharmacology, Inner Mongolia Minzu University, Tongliao 028000, China; anfengmao@126.com (F.A.); bmz3@163.com (M.B.); 2 Inner Mongolia Key Laboratory of Mongolian Medicine Pharmacology for Cardio-Cerebral Vascular System, Tongliao 028000, China; 3 Medical College, Inner Mongolia Minzu University, Tongliao 028000, China; 4 First Clinical Medical College, Inner Mongolia Minzu University, Tongliao 028000, China; lebao0908@163.com (Y.B.); xuanxr@126.com (X.X.); 5 College of Nursing, Inner Mongolia Minzu University, Tongliao 028000, China; tianjian0925@163.com; 6 Institute of Dementia, Inner Mongolia Minzu University, Tongliao 028000, China

**Keywords:** 1,8-cineole, Alzheimer’s disease, advanced glycation end products, tau hyperphosphorylation, β-amyloid

## Abstract

Advanced glycation end products (AGEs) are stable products produced by the reaction of macromolecules such as proteins, lipids or nucleic acids with glucose or other reducing monosaccharides, which can be identified by immunohistochemistry in the senile plaques and neurofibrillary tangles of Alzheimer’s disease (AD) patients. Growing evidence suggests that AGEs are important risk factors for the development and progression of AD. 1,8-cineole (CIN) is a monoterpenoid compound which exists in many plant essential oils and has been proven to have neuroprotective activity, but its specific effect and molecular mechanisms are not clear. In this study, AGEs-induced neuronal injury and intracerebroventricular-AGE animals as the possible models for AD were employed to investigate the effects of CIN on AD pathology as well as the molecular mechanisms involved both in vivo and in vitro. Our study demonstrated that CIN could ameliorate tau phosphorylation by down-regulating the activity of GSK-3β and reducing Aβ production by inhibiting the activity of BACE-1 both in vivo and in vitro. It is suggested that CIN has certain therapeutic value in the treatment of AD.

## 1. Introduction

Alzheimer’s disease (AD) is a chronic neurodegenerative disease with the characteristics of cognitive dysfunction and memory impairment. It is the most common cause of dementia. As population life expectancy increases, the number of people with dementia may triple by 2050 [1]. At present, only five drugs have been approved to treat AD by the FDA, mainly including cholinesterase inhibitors (donepezil, rivastigmine and galantamine), an NMDA receptor antagonist (memantine) and an anti-amyloid monoclonal antibody (aducanumab) [2]. It is urgent to develop safer and more effective drugs to prevent and treat AD.

The pathogenesis of AD is very complex and still eludes our comprehension. The main pathological features of AD are neurofibrillary tangles (NFTs) and deposited senile plaques (SPs) [3]. SPs are mainly due to the abnormal deposition of β-amyloid (Aβ) [4], and NFTs are mainly caused by the aggregation of hyperphosphorylated tau [5]. Accumulating evidence implies that advanced glycation end products (AGEs) in the brains of AD patients increase significantly [6,7], and immunohistochemical results reveal that AGEs are co-located with SPs and NFTs [8,9], suggesting that AGEs may be important molecules in the promotion of the process of AD. AGEs interact with the cell surface receptors of AGE (RAGE) to cause oxidative stress and produce a series of physiological and pathological reactions [10]. Oxidative stress can promote Aβ generation and tau hyperphosphorylation in quite a few ways. Oxidative stress induces DNA demethylation and histone acetylation, resulting in the activation of the AD-related gene transcription in the Aβ overproduction [11]. In a transgenic *Caenorhabditis elegans* model expressing Aβ1-42, oxidative stress preceded the fibrillar deposition of Aβ [12]. Oxidative stress is also an integral part of tau pathology. In cultured neurons isolated from an embryonic chick brain, oxidative stress induced tau hyperphosphorylation and led to the redistribution of tau away from neurites [13]. In the meantime, Aβ and hyperphosphorylated tau can further enhance oxidative stress, creating a critical vicious circle. Enhancing antioxidant enzyme activity can inhibit oxidative stress and protect nerve cells from Aβ-induced injury [14]. In a Drosophila model of human tauopathies, the antioxidant defense mechanism significantly changed the neurodegeneration, indicating that oxidative stress plays a causal role in neurotoxicity [15].

1,8-cineole (CIN) is a monoterpenoid compound found in many plant essential oils. Recent studies have demonstrated that CIN has a variety of pharmacological activities, including anti-inflammatory [16], antioxidant [17], anti-cholinesterase [18] activities and so on. CIN has shown potentially neuroprotective effects, but its molecular mechanisms are still unclear. Therefore, in the present experiments, we studied the effects of CIN on AGEs-induced AD pathology in vitro and in vivo as well as the molecular mechanisms involved.

## 2. Results

### 2.1. Effects of AGEs and CIN on SH-SY5Y Cell Viability

**Figure 1:**
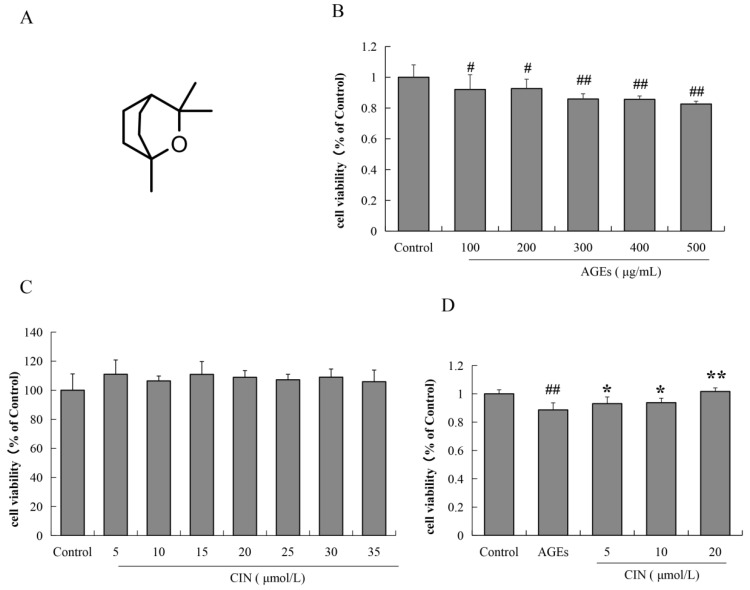
The chemical structure of CIN and the effects of AGEs and CIN on SH-SY5Y cell viability. (**A**) Chemical structure of CIN. (**B**) The MTT assay was used to detect the effect of AGEs on SH-SY5Y cell activity. (**C**) The MTT assay was used to detect the effect of CIN on SH-SY5Y cell activity. (**D**) The MTT assay was used to detect the effect of CIN on AGEs-induced SH-SY5Y cell activity. *n* = 6. ^#^ *p* < 0.05, ^##^ *p* < 0.01 vs. the control group; * *p* < 0.05, ** *p* < 0.01 vs. the model group.

SH-SY5Y cells were incubated with different concentrations of AGEs (100, 200, 300, 400 and 500 μg/mL) for 48 h, and the cell viability was estimated by MTT assay. The results show that after treatment with different concentrations of AGEs for 48 h, the viability of SH-SY5Y cells decreased significantly by different concentrations of AGEs for 48 h (*p* < 0.05 or *p* < 0.01), which was concentration-dependent (Figure 1B). We also used the MTT assay to detect whether CIN has a toxic effect on SH-SY5Y cells. As shown in Figure 1C, the concentration of CIN from 5 μmol/L to 35 μmol/L had no effect on cell viability. In the subsequent study, the concentration of AGEs used for SH-SY5Y cells was 500 μg/mL. We then detected the effect of CIN (5, 10 and 20 μmol/L) on AGEs-induced SH-SY5Y cell viability. It was shown that CIN significantly reversed the decrease in cell viability induced by AGEs (*p* < 0.05 or *p* < 0.01; Figure 1D).

### 2.2. CIN Prevented AGEs-Mediated Oxidative Damage

**Figure 2:**
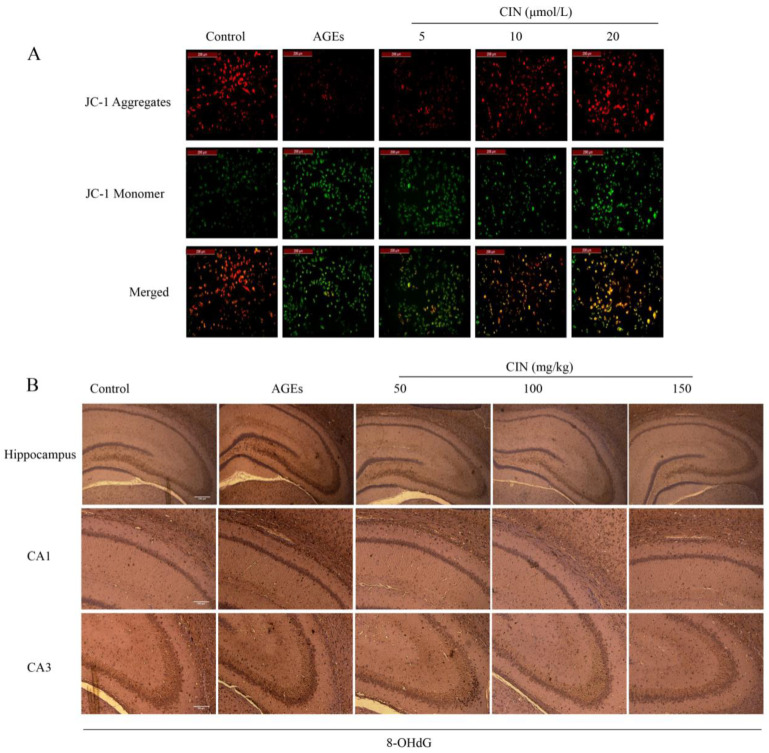
Effects of CIN on AGEs-induced oxidative damage. (**A**) Mitochondrial membrane potential was detected by JC-1 staining and viewed by fluorescence microscope (scale bar = 200 μm). (**B**) IHC staining of rat brain immunostained with antibody against 8-OHdG (scale bar, hippocampus group: 500 µm; CA1, CA3 group: 250 µm).

The decrease in mitochondrial membrane potential ∆Ψm is an important event of mitochondrial oxidative damage and early apoptosis. JC-1 is an ideal fluorescent probe to detect ∆Ψm. When ∆Ψm is high, the JC-1 probe forms a polymer in the matrix of mitochondria and emits red fluorescence; while ∆Ψm is low, the JC-1 probe exists in the form of a monomer and emits green fluorescence [19]. Strong green fluorescence appeared in the AGEs group, indicating that AGEs decreased the ∆Ψm of SH-SY5Y cells. The cells co-treated with different concentrations of CIN showed strong red fluorescence, indicating that ∆Ψm tended to be normal (Figure 2A).

8-OHdG, a sensitive marker of oxidative stress, is an oxidative product produced by reactive oxygen species (ROS) attacking DNA molecules. In order to estimate the oxidative damage in vivo, we injected AGEs into the rat hippocampus and detected the expression of 8-OHdG in hippocampal sections of each group by immunohistochemistry (IHC). Oxidative damage was significantly increased in the AGEs group, and CIN treatment prevented the accumulation of 8-OHdG (Figure 2B).

### 2.3. CIN Ameliorated AGEs-Induced Tau Hyperphosphorylation

**Figure 3:**
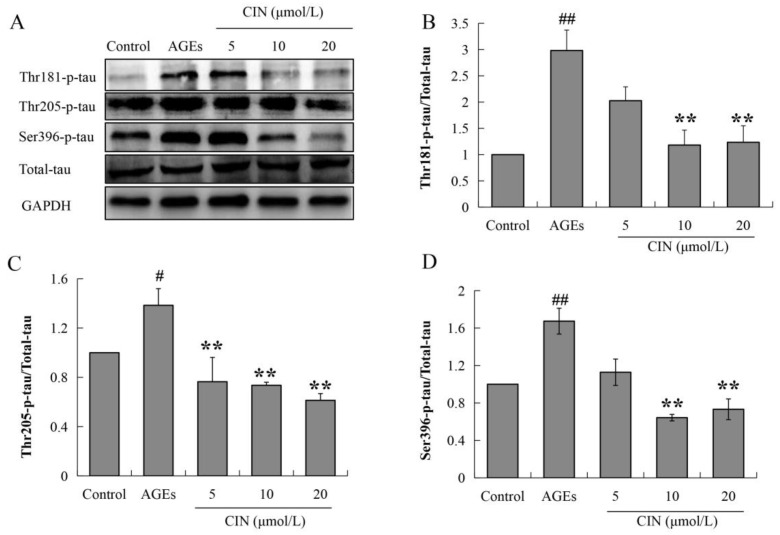
Effects of CIN on the tau hyperphosphorylation in vitro. (**A**–**D**) The protein levels of phosphorylated tau at thr181, thr205 and Ser396 sites in SH-SY5Y cells were detected by Western blot. *n* = 3. ^#^ *p* < 0.05, ^##^ *p* < 0.01 compared with the control group; ** *p* < 0.01 compared with the model group.

**Figure 4:**
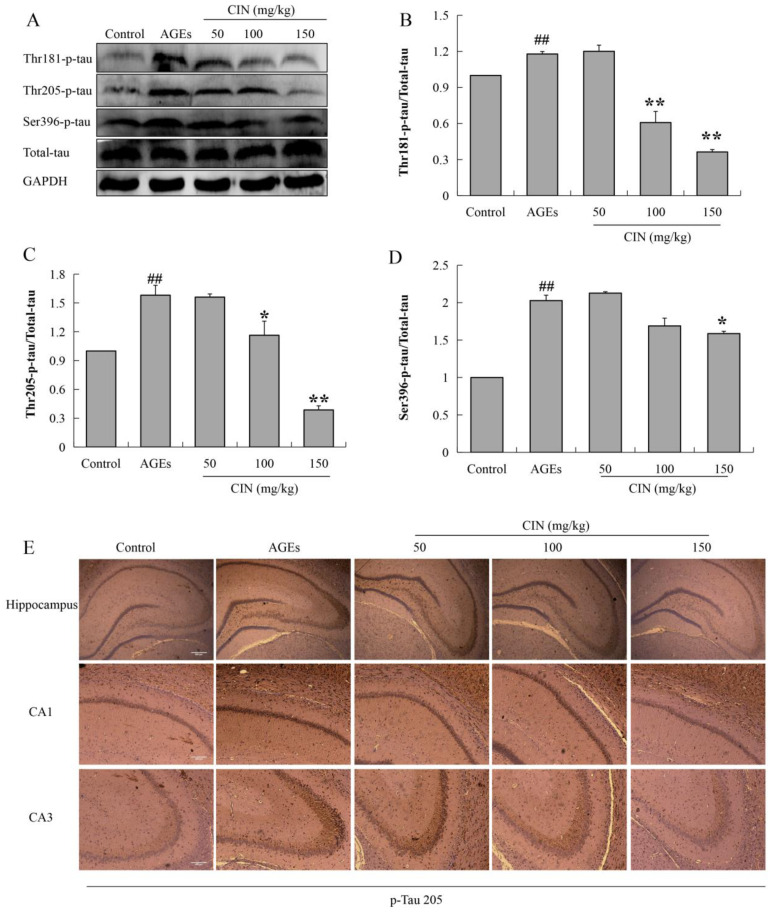
Effects of CIN on the tau hyperphosphorylation in vivo. (**A**–**D**) The protein levels of phosphorylated tau at thr181, thr205 and Ser396 sites in rat hippocampus were detected by Western blot. *n* = 8. (**E**) IHC staining of rat brain immunostained with antibody against thr205-phosphorylated tau (scale bar, hippocampus group: 500 µm; CA1, CA3 group: 250 µm). ^##^ *p* < 0.01 compared with the control group; * *p* < 0.05, ** *p* < 0.01 compared with the model group.

Hyperphosphorylated tau has neurotoxicity, resulting in neuronal synaptic damage and apoptosis. In order to explore the effect of CIN on AD pathology, we detected the phosphorylation level of the tau protein. The Western blot results showed that CIN could alleviate the abnormal phosphorylation levels of tau proteins at the thr205, thr181 and ser396 sites induced by AGEs both in vitro (*p* < 0.01; Figure 3) and in vivo (*p* < 0.05 or *p* < 0.01; Figure 4A–D). The IHC method was used to further determine the effect of CIN on the phosphorylation level of tau proteins at the thr205 site in the hippocampus. It was shown that tau was hyperphosphorylated at the thr205 site after the ICV-injected with AGEs and CIN treatment resulted in a decline of tau hyperphosphorylation (Figure 4E).

### 2.4. CIN Inhibited Activation of GSK-3β Induced by AGEs

**Figure 5:**
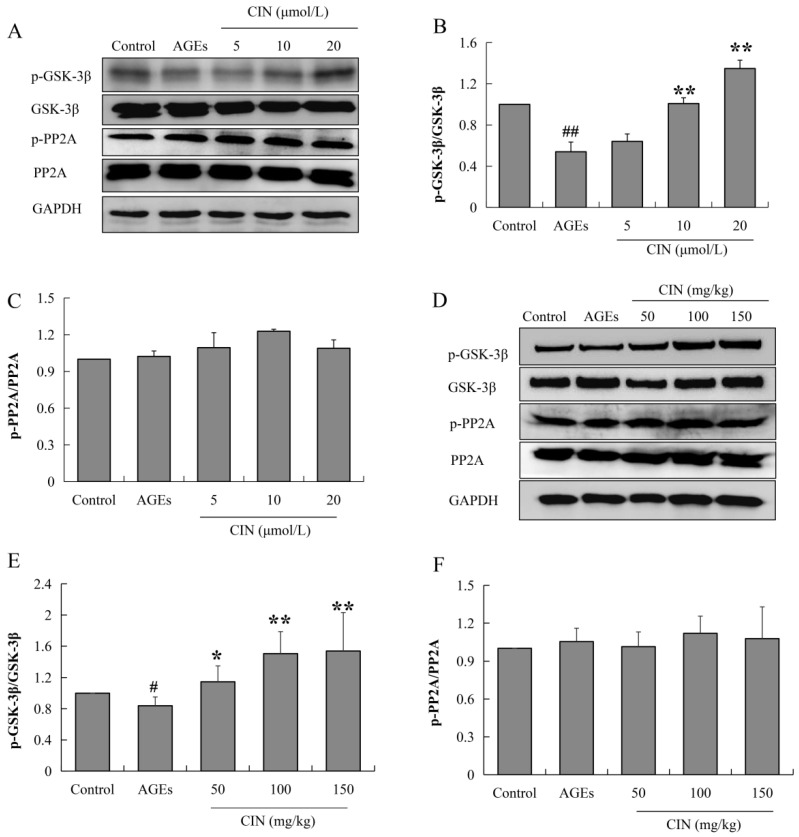
Effects of CIN on the activities of GSK-3β and PP2A in vitro and in vivo. (**A**–**C**) The protein levels of GSK-3β and PP2A in SH-SY5Y cells were detected by Western blot. *n* = 3. (**D**–**F**) The protein levels of GSK-3β and PP2A in rat hippocampus were detected by Western blot. *n* = 8. ^#^ *p* < 0.05, ^##^ *p* < 0.01 compared with the control group; * *p* < 0.05, ** *p* < 0.01 compared with the model group.

Tau phosphorylation is mainly regulated by the protein kinase and phosphatase activity. When phosphatase activity is inhibited or the protein kinase is over-activated, tau is hyperphosphorylated. Glycoase-3β (GSK-3β) is the most important protein kinase that regulates tau phosphorylation, and the enhancement of protein phosphatase 2A (PP2A) activity can inhibit abnormal tau phosphorylation. In order to investigate the mechanisms of CIN intervention in tau pathology, the activities of GSK-3β and PP2A were detected via Western blot. CIN inhibited the AGEs-induced overactivation of GSK-3β (*p* < 0.05 or *p* < 0.01), while the activity of PP2A was not affected both in vitro (Figure 5A–C) and in vivo (Figure 5D–F). This suggests that GSK-3β played a critical role in CIN preventing AGEs-induced tau phosphorylation.

### 2.5. CIN Decreased Aβ Production Induced by AGEs

**Figure 6:**
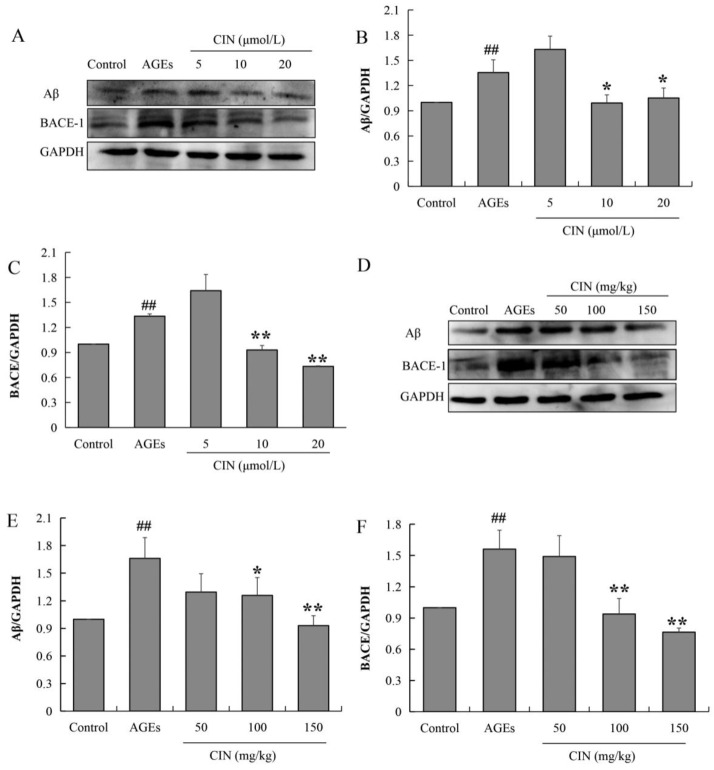
Effects of CIN on Aβ production and BACE expression in vitro and in vivo. (**A**–**C**) The protein levels of Aβ and BACE in SH-SY5Y cells were detected by Western blot. *n* = 3. (**D**–**F**) The protein levels of Aβ and BACE in rat hippocampus were detected by Western blot. *n* = 8. ^##^ *p* < 0.01 compared with the control group; * *p* < 0.05, ** *p* < 0.01 compared with the model group.

The aggregation and deposition of Aβ inhibit the function of neurons and induce synaptic dysfunction as well as memory impairment. Aβ peptides are derived from the β-amyloid precursor protein (APP), and the beta-site amyloid precursor protein cleaving enzyme-1 (BACE-1) is the rate-limiting enzyme to cleaved APP. To investigate the effect of CIN on AD pathology, we also detected the expressions of Aβ and BACE-1 via Western blot. The results show that AGE treatment increased the expression levels of Aβ and BACE-1, and CIN treatment resulted in a decline in AGEs-induced Aβ production by inhibiting BACE-1 both in vitro (*p* < 0.05 or *p* < 0.01; Figure 6A–C) and in vivo (*p* < 0.05 or *p* < 0.01; Figure 6D–F).

## 3. Discussion

There are many pathogeneses of AD, including acetylcholine hypothesis, Aβ hypothesis, tau protein hypothesis, neuroexcitatory toxicity hypothesis and so on, in which Aβ and abnormally phosphorylated tau protein are the main pathological features of AD. However, these mechanisms cannot fully explain the causes of various complex pathological phenomena in AD, which also makes the current drugs for the treatment of AD unable to reverse the disease, only to alleviate some symptoms, and the curative effect is limited. In recent years, the interconnections between type 2 diabetes mellitus (T2DM) and AD have attracted increasing attention. The Rotterdam Study showed that T2DM almost doubled the risk of dementia and AD [20]. A 32-year follow-up investigation confirmed that impaired insulin secretion, glucose intolerance and insulin resistance were all associated with a higher risk of AD [21]. AD is even called “type 3 diabetes”. AGEs are a group of stable and complex end products produced by Maillard series reactions between the free amino groups of proteins, amino acids, lipids or nucleic acids and the aldehyde groups of glucose or other reducing sugars under non-enzymatic conditions [22]. In the hyperglycemic state of T2DM, the accumulation of AGEs is accelerated and widely distributed in the pathological system of diabetes patients. It has recently become clear that AGEs also affect physiological aging and neurodegenerative diseases, such as AD.

CIN, also known as eucalyptol, is a major monoterpene in many aromatic plant essential oils. Recent studies have shown that CIN has a variety of pharmacological activities, including anti-apoptosis, antioxidant, anti-inflammatory activities and so on. In a mouse model of acute pancreatitis, CIN (200 mg/kg) could attenuate acute pancreatitis via an anti-inflammatory mechanism and by combating oxidative stress [23]. Linghu et al. reported that 1,8-cineole attenuated LPS-induced cell injury via modulating NF-κB [16]. The study of Ryu et al. showed that CIN had direct ROS scavenging activity [17]. A recent study revealed that CIN showed interesting selective inhibitory activity against acetylcholinesterase [18]. CIN is widely used in the pharmaceutical industry [24] and is also used as a condiment in food and cosmetics [25]. The oral administration of CIN (550 mg/kg bodyweight) in Wistar albino rats could restore their normal fasting blood glucose and blood lipid levels [26]. The protective effect of CIN (1.1 µg/mL) on vascular endothelial injury induced by hyperglycemia has also been reported [27]. Interestingly, CIN is a small lipophilic molecule that can easily penetrate the blood–brain barrier (BBB). Some studies have also confirmed that CIN possesses neuroprotective activity. CIN (10 μM and 25 μM) could inhibit ROS production in neurons and markedly enhance the activities of antioxidant enzymes including catalase, superoxide dismutase and glutathione peroxidase to decrease apoptosis induced by H2O2 [28]. In db/db mouse eyes, CIN (10 mg/kg) prevented apoptosis by inhibiting Aβ-mediated oxidative stress [29]. In PC12 cells, CIN (10 μM) reduced the levels of proinflammatory cytokines, namely ROS and NO, which were increased by the treatment of Aβ [30]. In our present study, we first explored the effect of CIN on AGEs-induced cytotoxicity, and the data showed that CIN significantly reversed the decrease in cell viability induced by AGEs. These results prompted us to explore the effect of CIN on AD pathology and further study the molecular mechanisms involved.

Aβ accumulation and tau hyperphosphorylation are major hallmarks of AD. AGEs accumulate in both intracellular and extracellular space and interact with RAGE, which is closely related to AD pathology. RAGE is also reported to mediate the transport of Aβ through the cell membrane of neurons and BBB. In the PD-hAPP mice transgenic model, the inhibition of the RAGE –ligand interaction suppressed the accumulation of Aβ in brain parenchyma [31]. The brains of RAGE-deficient mAPP mice displayed reduced Aβ by decreasing β- and γ-secretases activity and attenuated learning and memory impairment compared to mAPP mice [32]. In an AD mice model, high dietary AGEs accelerated Aβ deposition [33]. AGEs induce Aβ to flow through the BBB in a concentration- and time-dependent manner, accompanied by the increase in RAGE [34]. AGEs can also promote tau pathology. Even the short-term intake of dietary AGEs could lead to cognitive impairment, tau phosphorylation and neuroinflammation in aged ICR mice [35]. We also reported that injecting AGEs into the tails of mice could induce tau hyperphosphorylation in the brain [19]. Aminoguanidine, a blocker of AGEs formation, could effectively reverse tau hyperphosphorylation [36]. AGEs play a pivotal role in inducing oxidative stress by regulating a variety of cellular signaling pathways, which cause cell damage and death. It is reported that the AGE–RAGE axis regulated Aβ formation and tau phosphorylation via increased cathepsin B and asparagine endopeptidase [37]. AGEs caused the up-regulation of SOD, catalase and ROS, and induced Aβ secretion [38]. APP expression was up-regulated by AGEs, and the pretreatment of cells with an ROS inhibitor blocked the effects of AGEs [39]. It may be that AGEs/oxidative stress causes Aβ aggregation and tau hyperphosphorylation, while Aβ/tau pathology aggravates oxidative stress, promoting a vicious circle, and finally leading to AD. In the current study, we found that CIN could reverse AGEs-induced oxidative stress, tau hyperphosphorylation and Aβ production both in vivo and in vitro.

Tau phosphorylation is regulated by protein kinase and phosphatase. Protein kinase promotes AD by phosphorylating tau and eventually aggregating phosphorylated tau to form NFTs. On the contrary, protein phosphatase plays a completely opposite role in tau hyperphosphorylation. PP2A is the most important phosphatase regulating tau protein phosphorylation, and its enhanced activity can inhibit the abnormal phosphorylation of tau proteins [40]. GSK-3β, protein kinase A, calmodulin-dependent protein kinase II, mitogen-activated protein kinase and other protein kinases can phosphorylate tau proteins. Among them, GSK-3β is the most important protein kinase. If the activity of GSK-3β decreases, it can intervene in the occurrence of AD by inhibiting the hyperphosphorylation of tau [41]. In the brain of AD patients, the expression of the active form of GSK-3β was increased [42]. In APP/PS1 mouse models of AD mice, silencing GSK-3β significantly down-regulated the level of hyperphosphorylated tau and improved the memory ability of AD mice [43]. Meanwhile, GSK-3β is the key enzyme of glycogen synthesis which plays an important role in the regulation of glucose metabolism in DM. GSK-3β is a potential link between DM and AD. In our present study, in order to investigate the mechanisms of CIN on tau pathology, we further detected the activities of GSK-3β and PP2A both in vivo and in vitro. The results show that CIN could inhibit the activity of GSK-3β to reverse the AGEs-induced hyperphosphorylation of tau, while the activity of PP2A was not affected. Another pathological feature of AD is the abnormal deposition of Aβ. The sequential cleavage of APP by BACE-1 and γ-secretase results in the formation of Aβ. The inhibition of BACE-1 activity can reduce Aβ and SPs in AD-related brain regions and improve the damaged synaptic plasticity and learning and memory ability [44]. In order to investigate the mechanisms of CIN on Aβ pathology, we also detected BACE-1 activity both in vivo and in vitro, and the results suggest that CIN reduced Aβ production by inhibiting the activity of BACE-1.

## 4. Materials and Methods

### 4.1. Preparation of AGEs

Bovine serum albumin (BSA, 50 mg/mL; Biofroxx, Einhausen, Germany) and D-glucose (0.5 mol/L; Sigma, St. Louis, MO, USA) were dissolved in phosphate-buffered saline (PBS, 0.2 mol/L, pH 7.4), filtered and sterilized and incubated at 37 °C in darkness. After 90 days, the product was dialyzed in 0.01 mol/L PBS to remove free glucose and stored at −80 °C.

### 4.2. Cell Culture and Viability Assays

The SH-SY5Y cells were cultured in RPMI 1640 medium (Gibco Life Technologies, Grand Island, NY, USA) containing 10% fetal bovine serum (Gibco) and 1% penicillin–streptomycin mixture (Beyotime, Shanghai, China) for 24 h at 37 °C in a water-saturated 5% CO_2_ atmosphere. Cultured cells were treated with AGEs (100, 200, 300, 400 and 500 μg/mL), CIN (5, 10, 15, 20, 25 and 30 μmol/L; Sigma) or 500 μg/mL AGEs in the absence and presence of CIN (5, 10 and 20 μmol/L). After 48 h, the medium was removed, and MTT was added to each well to evaluate cell viability. The absorbance was measured at the wavelengths of 570 nm and 630 nm.

### 4.3. Animal and Treatments

Healthy SPF male Sprague Dawley rats (200 ± 20 g) were purchased from Changchun Yisi company (Changchun, China) and fed adaptively for one week before the experiment. The animals were kept in cages in a well-ventilated environment with a temperature of 23 ± 2 °C on a 12 h light/12 h dark cycle. Rats were randomly divided into the following groups (*n* = 12 in each group): control group, AGEs group, CIN low-dose group (50 mg/kg bodyweight), CIN medium-dose group (100 mg/kg bodyweight) and CIN high-dose group (150 mg/kg bodyweight). The rats in the control group were intracerebroventricularly (ICV) injected with sterile normal saline, and the rats in the AGEs group and CIN treatment groups were ICV-injected with AGEs (480 μg). After surgery, the rats of the CIN treatment groups were given CIN (50 mg/kg, 100 mg/kg, 150 mg/kg, respectively) once daily by gavage for 28 days, and the control groups and AGEs group were treated with saline in the same way daily. The ethics approval of this study was granted by the ethical committee of the medical faculty of Inner Mongolia Minzu University (M2016085).

### 4.4. Measurement of Mitochondrial Membrane Potential

Mitochondrial membrane potential was analyzed using JC-1 dye (Beyotime Biotechnology, Shanghai, China). After AGEs treatment in the absence and presence of CIN (5, 10 and 20 μmol/L) for 48 h, the SH-SY5Y cells were added with the solution containing JC-1 fluorescent probe and fully contacted for 30 min at 37 °C. The fluorescence of JC-1 probe in each group was detected by fluorescence microscope (Leica, Solms, Germany).

### 4.5. Immunohistochemistry

The brain tissue of rats was fixed in 4% paraformaldehyde solution overnight and embedded in paraffin. Serial sections of 5 μm thickness were cut and incubated overnight with primary antibodies against thr205-phosphorylated tau antibody (Santa Cruz Biotechnology, Santa Cruz, CA, USA) or 8-OHdG antibody (Santa Cruz Biotechnology) at 4 °C. After washing with PBS, the sections were incubated with HRP-conjugated secondary antibody, and immunological complexes were detected by Diaminobenzidine Tetrahydrochloride (Zymed, South San Francisco, CA, USA).

### 4.6. Western Blotting Analysis

SH-SY5Y cells or rat hippocampal tissue were lysed in RIPA buffer containing protease inhibitor and phosphatase inhibitor. The proteins samples were separated by 10% or 12% sodium dodecyl sulfate polyacrylamide gel electrophoresis and transferred to PVDF membranes (Millpore, Temecula, CA, USA). Then, the membranes were incubated with primary antibodies against ser396-phosphorylated tau-(sc-32275), PP2A-(sc-80665), p-PP2A-(sc-271903) antibodies (1:1000; Santa Cruz Biotechnology), thr205-phosphorylated tau-(49561), thr181-phosphorylated tau-(12885), total-tau-(4019), p-GSK-3β-(5558), GSK-3β-(12456), GAPDH-(5174), BACE-1-(5606) and Aβ-(8243) antibodies (1:1000; Cell Signaling Technology, Danvers, MA, USA) at 4 °C. After overnight incubation, the membranes were incubated with horseradish peroxidase-labeled secondary antibody at room temperature for 1 h, and immunological complexes were detected by enhanced chemiluminescence reagents (Pierce, Rockford, IL, USA). The band intensity analysis of Western blot was calculated by image J software.

### 4.7. Statistical Analysis

All data were expressed as mean ± standard error of the mean and statistically analyzed by SPSS 17.0 software. One-way ANOVA followed by Tukey’s as a post hoc hypothesis testing procedure was used for comparison between groups. *p* value < 0.05 was considered to be statistically significant.

## 5. Conclusions

In summary, our present findings suggest that AGEs can induce oxidative stress, tau hyperphosphorylation and Aβ production. CIN can regulate tau phosphorylation by down-regulating the activity of GSK-3β and reduce Aβ production by inhibiting the activity of BACE-1 both in vivo and in vitro. It is suggested that CIN has certain therapeutic value in the treatment of AD, especially DM-related AD. Although oxidative stress was incorporated in this study, the effect of CIN on mitochondrial dysfunction needs to be more extensively explored.

## References

[1] World Alzheimer Report 2018. https://www.alzint.org/u/WorldAlzheimerReport2018.pdf.

[2] Karlawish J., Grill J.D. (2021). The approval of Aduhelm risks eroding public trust in Alzheimer research and the FDA. Nat. Rev. Neurol..

[3] Lane C.A., Hardy J., Schott J.M. (2018). Alzheimer’s disease. Eur. J. Neurol..

[4] Reiss A.B., Arain H.A., Stecker M.M., Siegart N.M., Kasselman L.J. (2018). Amyloid toxicity in Alzheimer’s disease. Rev. Neurosci..

[5] Naseri N.N., Wang H., Guo J., Sharma M., Luo W. (2019). The complexity of tau in Alzheimer’s disease. Neurosci. Lett..

[6] Beeri M.S., Uribarri J., Cai W., Buchman A.S., Haroutunian V. (2020). Human Brain and Serum Advanced Glycation End Products are Highly Correlated: Preliminary Results of Their Role in Alzheimer Disease and Type 2 Diabetes. Endocr. Pract..

[7] Tabara Y., Yamanaka M., Setoh K., Segawa H., Kawaguchi T., Kosugi S., Nakayama T., Matsuda F., Nagahama Study Group (2020). Advanced Glycation End Product Accumulation is Associated with Lower Cognitive Performance in an Older General Population: The Nagahama Study. J. Alzheimers Dis..

[8] Sasaki N., Toki S., Chowei H., Saito T., Nakano N., Hayashi Y., Takeuchi M., Makita Z. (2001). Immunohistochemical distribution of the receptor for advanced glycation end products in neurons and astrocytes in Alzheimer’s disease. Brain Res..

[9] Valente T., Gella A., Fernàndez-Busquets X., Unzeta M., Durany N. (2010). Immunohistochemical analysis of human brain suggests pathological synergism of Alzheimer’s disease and diabetes mellitus. Neurobiol. Dis..

[10] Srikanth V., Maczurek A., Phan T., Steele M., Westcott B., Juskiw D., Münch G. (2011). Advanced glycation endproducts and their receptor RAGE in Alzheimer’s disease. Neurobiol. Aging..

[11] Gu X., Sun J., Li S., Wu X., Li L. (2013). Oxidative stress induces DNA demethylation and histone acetylation in SH-SY5Y cells: Potential epigenetic mechanisms in gene transcription in Aβ production. Neurobiol. Aging.

[12] Drake J., Link C.D., Butterfield D.A. (2003). Oxidative stress precedes fibrillar deposition of Alzheimer’s disease amyloid beta-peptide (1-42) in a transgenic Caenorhabditis elegans model. Neurobiol. Aging.

[13] Beyrent E., Gomez G. (2020). Oxidative stress differentially induces tau dissociation from neuronal microtubules in neurites of neurons cultured from different regions of the embryonic Gallus domesticus brain. J. Neurosci. Res..

[14] Li Q., Cui J., Fang C., Liu M., Min G., Li L. (2017). S-Adenosylmethionine Attenuates Oxidative Stress and Neuroinflammation Induced by Amyloid-β Through Modulation of Glutathione Metabolism. J. Alzheimers Dis..

[15] Dias-Santagata D., Fulga T.A., Duttaroy A., Feany M.B. (2007). Oxidative stress mediates tau-induced neurodegeneration in Drosophila. J. Clin. Investig..

[16] Linghu K.G., Wu G.P., Fu L.Y., Yang H., Li H.Z., Chen Y., Yu H., Tao L., Shen X.C. (2019). 1,8-Cineole Ameliorates LPS-Induced Vascular Endothelium Dysfunction in Mice via PPAR-γ Dependent Regulation of NF-κB. Front. Pharmacol..

[17] Ryu S., Park H., Seol G.H., Choi I.Y. (2014). 1,8-Cineole ameliorates oxygen-glucose deprivation/reoxygenation-induced ischaemic injury by reducing oxidative stress in rat cortical neuron/glia. J. Pharm. Pharmacol..

[18] Panamito M.F., Bec N., Valdivieso V., Salinas M., Calva J., Ramírez J., Larroque C., Armijos C. (2021). Chemical Composition and Anticholinesterase Activity of the Essential Oil of Leaves and Flowers from the Ecuadorian Plant *Lepechinia paniculata* (Kunth) Epling. Molecules.

[19] An F.M., Chen S., Xu Z., Yin L., Wang Y., Liu A.R., Yao W.B., Gao X.D. (2015). Glucagon-like peptide-1 regulates mitochondrial biogenesis and tau phosphorylation against advanced glycation end product-induced neuronal insult: Studies in vivo and in vitro. Neuroscience.

[20] Ott A., Stolk R.P., van Harskamp F., Pols H.A., Hofman A., Breteler M.M. (1999). Diabetes mellitus and the risk of dementia: The Rotterdam Study. Neurology.

[21] Rönnemaa E., Zethelius B., Sundelöf J., Sundström J., Degerman-Gunnarsson M., Berne C., Lannfelt L., Kilander L. (2008). Impaired insulin secretion increases the risk of Alzheimer disease. Neurology.

[22] Sato T., Shimogaito N., Wu X., Kikuchi S., Yamagishi S., Takeuchi M. (2006). Toxic advanced glycation end products (TAGE) theory in Alzheimer’s disease. Am. J. Alzheimer’s Dis. Other Demen..

[23] Lima P.R., de Melo T.S., Carvalho K.M., de Oliveira Í.B., Arruda B.R., de Castro Brito G.A., Rao V.S., Santos F.A. (2013). 1,8-cineole (eucalyptol) ameliorates cerulein-induced acute pancreatitis via modulation of cytokines, oxidative stress and NF-κB activity in mice. Life Sci..

[24] Santos F.A., Rao V.S. (2000). Antiinflammatory and antinociceptive effects of 1,8-cineole a terpenoid oxide present in many plant essential oils. Phytother. Res..

[25] Caputi L., Aprea E. (2011). Use of terpenoids as natural flavouring compounds in food industry. Recent Pat. Food Nutr. Agric..

[26] Paul K., Bhattacharjee P., Chatterjee N., Pal T.K. (2019). Nanoliposomes of Supercritical Carbon Dioxide Extract of Small Cardamom Seeds Redresses Type 2 Diabetes and Hypercholesterolemia. Recent Pat. Biotechnol..

[27] Peng J., Jiang Z., Wu G., Cai Z., Du Q., Tao L., Zhang Y., Chen Y., Shen X. (2021). Improving protection effects of eucalyptol via carboxymethyl chitosan-coated lipid nanoparticles on hyperglycaemia-induced vascular endothelial injury in rats. J. Drug Target..

[28] Porres-Martínez M., González-Burgos E., Carretero M.E., Gómez-Serranillos M.P. (2016). In vitro neuroprotective potential of the monoterpenes α-pinene and 1,8-cineole against H_2_O_2_-induced oxidative stress in PC12 cells. Z. Naturforsch. C. J. Biosci..

[29] Kim D.Y., Kang M.K., Lee E.J., Kim Y.H., Oh H., Kim S.I., Oh S.Y., Na W., Kang Y.H. (2020). Eucalyptol Inhibits Amyloid-β-Induced Barrier Dysfunction in Glucose-Exposed Retinal Pigment Epithelial Cells and Diabetic Eyes. Antioxidants.

[30] Khan A., Vaibhav K., Javed H., Tabassum R., Ahmed M.E., Khan M.M., Khan M.B., Shrivastava P., Islam F., Siddiqui M.S. (2014). 1,8-cineole (eucalyptol) mitigates inflammation in amyloid Beta toxicated PC12 cells: Relevance to Alzheimer’s disease. Neurochem. Res..

[31] Deane R., Du Yan S., Submamaryan R.K., LaRue B., Jovanovic S., Hogg E., Welch D., Manness L., Lin C., Yu J. (2003). RAGE mediates amyloid-beta peptide transport across the blood-brain barrier and accumulation in brain. Nat. Med..

[32] Fang F., Yu Q., Arancio O., Chen D., Gore S.S., Yan S.S., Yan S.F. (2018). RAGE mediates Aβ accumulation in a mouse model of Alzheimer’s disease via modulation of β- and γ-secretase activity. Hum. Mol. Genet..

[33] Lubitz I., Ricny J., Atrakchi-Baranes D., Shemesh C., Kravitz E., Liraz-Zaltsman S., Maksin-Matveev A., Cooper I., Leibowitz A., Uribarri J. (2016). High dietary advanced glycation end products are associated with poorer spatial learning and accelerated Aβ deposition in an Alzheimer mouse model. Aging Cell.

[34] Chen F., Ghosh A., Hu M., Long Y., Sun H., Kong L., Hong H., Tang S. (2018). RAGE-NF-κB-PPARγ Signaling is Involved in AGEs-Induced Upregulation of Amyloid-β Influx Transport in an In Vitro BBB Model. Neurotox. Res..

[35] Yang S., Zhou H., Wang G., Zhong X.H., Shen Q.L., Zhang X.J., Li R.Y., Chen L.H., Zhang Y.H., Wan Z. (2020). Quercetin is protective against short-term dietary advanced glycation end products intake induced cognitive dysfunction in aged ICR mice. J. Food Biochem..

[36] Li X.H., Xie J.Z., Jiang X., Lv B.L., Cheng X.S., Du L.L., Zhang J.Y., Wang J.Z., Zhou X.W. (2012). Methylglyoxal induces tau hyperphosphorylation via promoting AGEs formation. Neuromolecular Med..

[37] Batkulwar K., Godbole R., Banarjee R., Kassaar O., Williams R.J., Kulkarni M.J. (2018). Advanced Glycation End Products Modulate Amyloidogenic APP Processing and Tau Phosphorylation: A Mechanistic Link between Glycation and the Development of Alzheimer’s Disease. ACS Chem. Neurosci..

[38] Wang L., Yu C.J., Liu W., Cheng L.Y., Zhang Y.N. (2011). Rosiglitazone protects neuroblastoma cells against advanced glycation end products-induced injury. Acta Pharmacol. Sin..

[39] Ko S.Y., Lin Y.P., Lin Y.S., Chang S.S. (2010). Advanced glycation end products enhance amyloid precursor protein expression by inducing reactive oxygen species. Free Radic. Biol. Med..

[40] Liu F., Grundke-Iqbal I., Iqbal K., Gong C.X. (2005). Contributions of protein phosphatases PP1, PP2A, PP2B and PP5 to the regulation of tau phosphorylation. Eur. J. Neurosci..

[41] Hur E.M., Zhou F.Q. (2010). GSK3 signalling in neural development. Nat. Rev. Neurosci..

[42] Leroy K., Yilmaz Z., Brion J.P. (2007). Increased level of active GSK-3beta in Alzheimer’s disease and accumulation in argyrophilic grains and in neurones at different stages of neurofibrillary degeneration. Neuropathol. Appl. Neurobiol..

[43] Bian H., Bian W., Lin X., Ma Z., Chen W., Pu Y. (2016). RNA Interference Silencing of Glycogen Synthase Kinase 3β Inhibites Tau Phosphorylation in Mice with Alzheimer Disease. Neurochem. Res..

[44] Das B., Yan R. (2017). Role of BACE1 in Alzheimer’s synaptic function. Transl. Neurodegener..

